# Co-infection with hepatitis B virus among tuberculosis patients is associated with poor outcomes during anti-tuberculosis treatment

**DOI:** 10.1186/s12879-018-3192-8

**Published:** 2018-07-03

**Authors:** Lubiao Chen, Dujing Bao, Lin Gu, Yurong Gu, Liang Zhou, Zhiliang Gao, Yuehua Huang

**Affiliations:** 1 Department of Infectious Diseases, The Third Affiliated Hospital of Sun Yat-sen University, 600, Tianhe Road, Guangzhou, 510630 China; 2 Guangdong Provincial Key Laboratory of Liver Disease Research, The Third Affiliated Hospital of Sun Yat-sen University, 600, Tianhe Road, Guangzhou, 510630 China

**Keywords:** Tuberculosis, Hepatitis B virus, Drug-induced liver injury, Liver failure, Clinical outcome

## Abstract

**Background:**

Tuberculosis (TB) and chronic Hepatitis B virus (HBV) infection are common in China. Fist-line anti-TB medications often produce drug-induced liver injury (DILI). This study sought to investigate whether TB patients with chronic HBV co-infection are more susceptible to liver failure and poor outcomes during anti-TB treatment.

**Methods:**

Eighty-four TB patients developed DILI during anti-TB treatment and were enrolled, including 58 with chronic HBV co-infection (TB-HBV group) and 26 with TB mono-infection (TB group). Clinical data and demographic characteristics were reviewed. The severity of DILI and incidences of liver failure and death were compared. Risk factors of clinical outcomes were defined.

**Results:**

The patterns of DILI were similar in both groups. Compared with patients in the TB group, patients in the TB-HBV group who did not receive anti-HBV therapy before anti-TB treatment were more susceptible to Grade-4 severity of DILI (36.2% vs. 7.7%, *P =* 0.005), liver failure (67.2% vs. 38.5%, *P =* 0.013) and poor outcomes (37.9% vs. 7.7%, *P =* 0.005). Age > 50 years (48.1% vs. 22.6%, *P =* 0.049), cirrhosis (50.0% vs. 15.4%, *P =* 0.046) and total bilirubin > 20 mg/dl (51.6% vs. 14.8%, *P =* 0.005) were independent risk factors for the rate of death in the TB-HBV group, and HBV DNA > 20,000 IU/ml had borderline significance (44.1% vs. 20.8%, *P =* 0.081). In the TB-HBV group, nucleos(t)ide analogues as rescue therapy were not able to reduce short-term death (33.3% vs. 36.8%, *P =* 0.659) once liver failure had occurred.

**Conclusions:**

Patients on anti-TB therapy with chronic HBV co-infection are more susceptible to developing liver failure and having poor outcomes during anti-TB treatment. Regular monitoring of liver function and HBV DNA level is mandatory. Anti-HBV treatment should be considered in those with high viral levels before anti-TB treatment.

**Electronic supplementary material:**

The online version of this article (10.1186/s12879-018-3192-8) contains supplementary material, which is available to authorized users.

## Background

Tuberculosis (TB) and chronic hepatitis B virus (HBV) infections are two major global health problems. In 2016, there were an estimated 10.4 million new TB cases worldwide and 1.67 million TB deaths according to the WHO global TB report in 2017 [1]. According to the 5th national tuberculosis epidemiological survey in 2010, China had a high incidence of active pulmonary TB (459/100,000 in the population aged > 15 years old) [2]. Although programmed HBV vaccination for newborns has been conducted since 1992, there were still an estimated 93 million chronic HBV infections and a prevalence of 7.8% hepatitis B virus surface antigen (HBsAg)-positive people [3]. The incidence of TB/HBV co-infection remains unknown. The treatment for TB patients co-infected with HBV is a challenging health problem in China as well as worldwide.

At present, isoniazid (INH), rifampin (RFP), pyrazinamide (PZA) and ethambutol (EMB) are first-line medications for those with drug sensitive tuberculosis infection in their initial intensive anti-TB treatment. Hepatotoxicity with INH, RFP and PZA has been reported more frequently, with an incidence ranging from 3 to 28% for idiosyncratic and intrinsic toxic reactions [4–7]. HBV is a non-cellular pathogen, but active HBV replication can lead to immune liver injury and even liver failure, especially in adults [8]. Although nucleos(t)ide analogue (NA) anti-HBV drugs including lamivudine (LAM), adefovir (ADV), telbivudine (LDT), entecavir (ETV) and tenofovir have been available, TB patients with chronic HBV co-infection receiving anti-HBV treatment prior to anti-TB treatment are still an overlooked problem in current general clinical practice.

Hepatotoxicity of anti-TB medications and immune liver injury caused by HBV replication exacerbate liver dysfunction [9, 10]. In this study, we conducted a retrospective investigation to analyze whether there were any differences in clinical characteristics and risk factors predicting differences in short-term prognoses between TB mono-infected patients and TB/HBV co-infected patients. We also aimed to focus on the importance of regular monitoring of TB patients receiving anti-TB treatment for their liver function and HBV DNA levels and to emphasize that anti-HBV treatment by NAs should be recommended before anti-TB treatment for those with active HBV replication to reduce the incidence of liver injury and liver failure.

## Methods

### Patients and data collection

Inpatients with TB who had undergone anti-TB drug-induced liver injury (DILI) before admission to the Third Affiliated Hospital of Sun Yat-sen University between March 2009 and September 2016 and had received anti-TB treatment in other hospitals were included. A total of 84 patients (Han Chinese) were enrolled, of whom 58 were HBsAg-positive (TB-HBV group) and the remaining 26 were HBsAg-negative (TB group). HAV, HCV, HDV, HEV and HIV co-infection, auto-immune hepatitis, liver cancer and Wilson’s diseases were excluded. All patients were followed for 6 months (180 days) after their liver injuries were diagnosed or until death occurred. This study was approved by the Ethical Committee of the Third Affiliated Hospital of the Sun Yat-sen University.

Medical records were reviewed in detail. Prior to anti-TB treatment, liver functions were normal according to their local hospital’s laboratory reference ranges in all patients. After initiating anti-TB treatment, they suffered from liver injury with different symptoms including fatigue, nausea, anorexia, vomiting, abdominal distention, pruritus, dark urine and jaundice and were therefore transported to our hospital. During the inpatient ward and follow-up periods, information on demographics (age, sex), serum HBV markers (HBsAg, HBV DNA levels), hepatic panel (including aspartate aminotransferase [AST], alanine aminotransferase [ALT], total bilirubin [TBIL], etc.), complete blood count, coagulation index (prothrombin time [PT] and activity [PTA], international normalized ratio [INR]) and clinical complications were recorded. Medications used and influences related to anti-TB drugs during treatment were reviewed.

### Inclusion criteria and causality assessments

Patients with abnormalities in liver function tests including any one of the following [11] during anti-TB treatment were enrolled: (i) ALT ≥5 fold elevation above the upper limit of normal (ULN); (ii) ALP ≥2 ULN in the absence of known bone pathology; (iii) TBIL > 2 ULN and ALT ≥3 ULN. Causality assessments were conducted by applying the RUCAM (Roussel Uclaf Causality Assessment Method) system [12] and patients with scoring ≥3 were included.

### Definitions

According to the American College of Gastroenterology clincal guideline [13] and LiverTox definition [14], R-value (ALT/ULN÷ALP/ULN) was calculated based on patient’s first liver function test after admission to our hospital. Patterns of DILI were defined as follows: hepatocellular pattern as ALT levels ≥3 ULN and R-value ≥5; cholestatic pattern as ALP ≥ 2 ULN and R-value ≤2; mixed pattern as ALT ≥3 ULN and ALP ≥2 ULN with R-value between 2 and 5. A new grading system [11] was applied to classify the severity of liver injury from the time of study entry to the end of 6 month follow up. Besides elevated ALT or ALP levels reaching criteria for DILI (ALT ≥3 ULN, ALP ≥ 2 ULN), severities of DILI were defined as: Grade-1 if TBIL was < 2 ULN; Grade-2 if TBIL was ≥2 ULN; Grade-3 if TBIL was ≥2 ULN including any one of INR ≥1.5, ascites or hepatic encephalopathy (HE); Grade-4 if a patient died or underwent liver transplantation because of a DILI event.

Liver failure was defined as serum TBIL levels > 5 ULN and INR > 1.5 or PTA < 40% with or without overt hepatic encephalopathy (HE) [15]. HE was not mandatory for the diagnosis of liver failure in this retrospective study.

Better outcome was defined as recovery (ALT < 2 ULN, TBIL < 2 ULN, INR returned to normal range) or improvement (ALT, TBIL and INR decreased) in the 6 months after DILI was diagnosed. Poor outcome was defined as exacerbation (increase in TBIL and INR or development of irreversible complications) or death in 6 months after the diagnosis of DILI.

### Laboratory assays

All laboratory assays were conducted in the Department of Laboratory at the Third Affiliated Hospital of Sun Yat-sen University. Liver function tests were measured using a Hitachi 7180 automatic analyzer (Hitachi Corporation, Japan) (Normal reference range: ALT 3–35 U/L, AST 15–40 U/L, TBIL 4.0–23.9 umol/L, GGT 10–60 U/L, ALP 45–125 U/L). Full blood count was assayed using a SYSMEX XE5000 system (Sysmex Corporation, Japan), and coagulation tests were performed with a STAGO STR automatic analyzer (Stago group, France). Serum HBV antigens and antibodies were detected by time-resolved fluorescence immunoassay (Sym-bio Corporation, China). HBV DNA levels were quantified by an ABI 3700 thermal cycler (Applied Biosystem, USA) using Da’an reagents (Da’an Corporation, China) (detection range from 100 IU/ml to 1.70E + 8 IU/ml).

### Diagnosis of liver cirrhosis

All patients received the Doppler ultrasonographic exmination (MylabTwice, Esoate, Genoa, Italy) after admission to our hospital. The examination was performed by the experienced ultrasound physician. An ultrasosographic scoring system consisting of liver surface (score: 1, smooth surface; 2, mild uneven or waveform surface; 3, undulated or irregular nodular surface), liver parenchyma (score: 1, homogeneous appearance; 2, heterogeneous appearance with fine scattered hypoechoic and hyperechoic areas; 3, coarse liver with irregular pattern), hepatic vascular structure (score: 1, smooth vessel wall; 2, obscured vessel with normal diameter; 3, irregular and narrowed vessel), and splenic size (score: 1, spleen size index [the product of the oblique and diagonal diameters] of less than 20 cm^2^; 2, larger spleen) was applied to describe the severity of hepatic paranchymal damage [16]. Score ≥ 7 was used to make the diagnosis of liver cirrhosis.

No patient received liver biopsy for fibrosis analysis. Only 9 patients received liver transient elasticity measurements by FibroScan (Echosens, Paris, France) because this technique was not introduced to our hospital before 2013.

### Statistical analysis

All statistical analyses were performed using SPSS 22.0 (SPSS Inc. Chicago, USA). Quantitative data with normal distributions were denoted as the means ± standard deviations. Student’s *t* test was used to compare the differences. Abnormally distributed quantitative data were denoted as medians (interquartile ranges) and Mann-Whitney *U* test was performed to compare the differences. Categorical variables were compared using Fisher’s exact test or chi-square test, as appropriate. The proportion of surviving patients in different groups was analyzed using the Kaplan-Meier method, and comparisons of the differences were performed using log-rank test. The risk factors of liver failure and poor outcomes were investigated by logistic regression analysis. The odds ratio (OR) and 95% confidence interval (95%CI) were calculated for each variable included. A two-tailed *P*-value of < 0.05 was considered statistically significant.

## Results

### Patient characteristics

The clinical and laboratory characteristics of all patients are not shown (more details seen in Additional file 1). Sixty five patients (77.4%) had pulmonary TB infection. There were more males in the TB-HBV group than in the TB group (91.4% vs. 53.9%, *P =* 0.000). Roughly half of the patients in the TB-HBV group had liver cirrhosis (55.2% vs. 7.7%, *P =* 0.000) diagnosed by the experienced ultrasound physician. However, platelet (PLT) counts were lower in TB-HBV group than in TB group, although both groups were within the normal range. Otherwise, there were no significant differences in mean age, alcohol intake (> 40 g/d), serum creatinine level, or hospital stays between the two groups. Patients in the TB-HBV group were more susceptible to Grade-4 severity of DILI (36.2% vs. 7.7%, *P =* 0.005), liver failure (67.2% vs. 38.5%, *P =* 0.013) and poor outcomes (37.9% vs. 7.7%, *P =* 0.005) compared with patients in the TB group. Maximum TBIL level in TB-HBV group was significantly higher than that in TB group (Median [μmol/L]: 391.4 vs. 145.8, *P =* 0.007).

Most patients received first-line anti-TB medications, with doses including INH 0.3 g/d, RFP 0.45 g–0.6 g/d, PZA 1.5 g/d, and/or EMB 0.75 g–1.0 g/d, and a few of them received INH 0.3 g/d and ofloxacin or levofloxacin 0.6 g/d. According to the first time liver function tests after admission to our hospital, 56 (66.7%), 14 (16.7%) and 14 (16.7%) patients were classified as hepatocellular, cholestic and mixed patterns of DILI respectively. Nevertheless, the distributions of the DILI patterns were similar regardless of HBV co-infection. Compared to the TB group, the latency of DILI in the TB-HBV group was longer but not statistically significant (Median [days]: 84.0 vs. 79.5, *P =* 0.462).

### Liver failure during anti-TB treatment

**Fig. 1:**
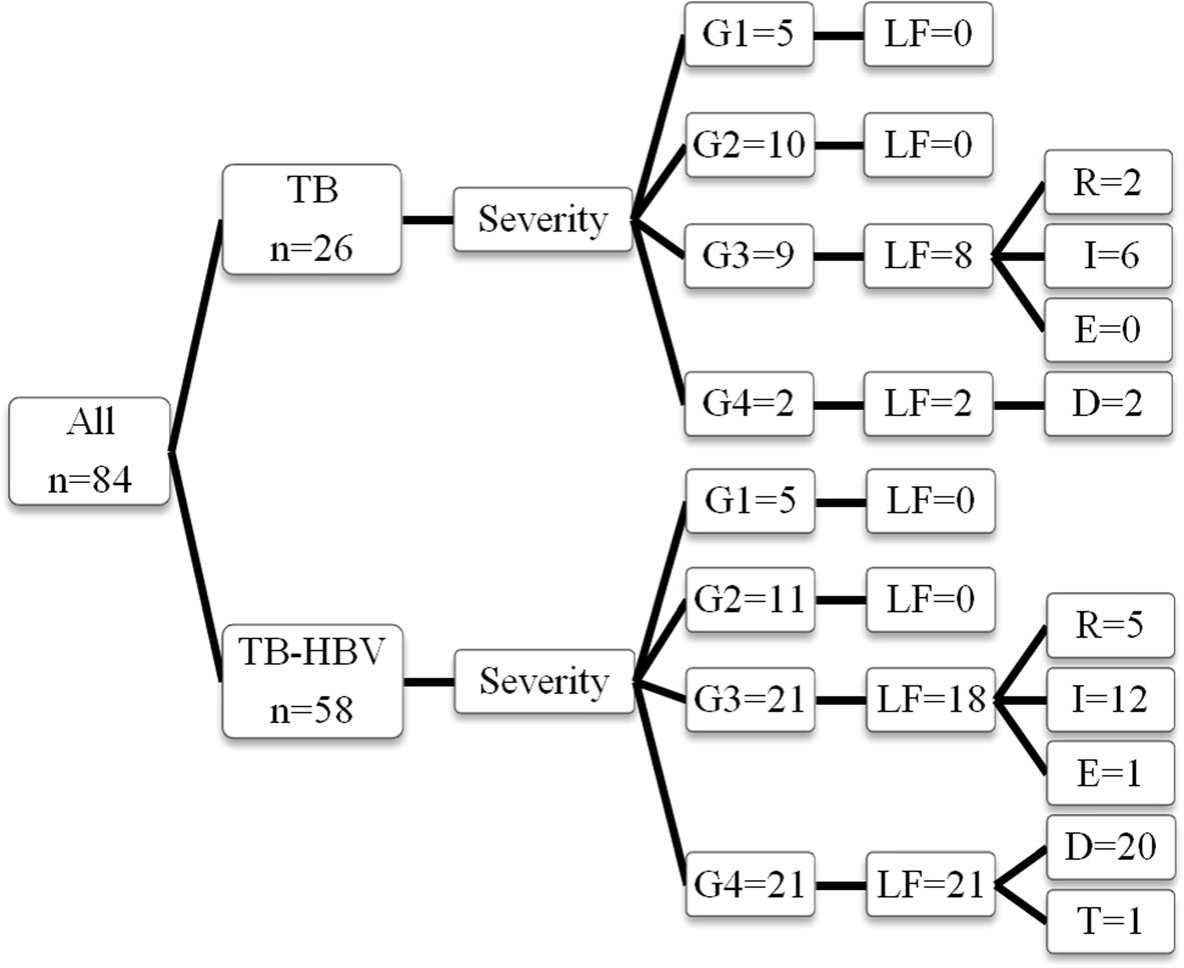
Liver failure in the two groups and their clinical outcomes. Ten (38.5%) patients in the TB group developed liver failure compared to 39 (67.2%) in the TB-HBV group (*P =* 0.013). In the TB-HBV group, 20 patients died and 1 received orthotopic liver transplantation, which was classified as Grade-4 severity of DILI, while only 2 patients with Grade-4 severity of DILI in the TB group died. Abbreviations: TB, tuberculosis; HBV, hepatitis B virus; G, grade; LF, liver failure; R, recovered; I, improved; E, exacerbated; D, died; T, liver transplantation

As shown in Fig. 1, the patients in the two groups with different severity grading of DILI developed liver failure and experienced different clinical outcomes. More patients in the TB-HBV group developed Grade-4 severity of DILI (36.2% vs. 7.7%, *P =* 0.005) (20 patients died and 1 patient received orthotopic liver transplantation [OLT]) and liver failure (67.2% vs. 38.5%, *P =* 0.013) than in the TB group. In the TB group, only two patients developed Grade-4 severity of DILI and 10 patients progressed to liver failure, while 21 and 39 patients in TB-HBV group. Both univariate and multivariate analyses indicated that the factors related to liver failure included HBV co-infection and cirrhosis in all patients (Table 1). Analyses of the TB-HBV group indicated that only cirrhosis was an independent risk for liver failure (Table 2).

**Table 1:** Analyses of risk factors for the incidence of liver failure in all patients (*n* = 84)

Factors	Univariate analysis			Multivariate analysis		
	OR	95%CI	*P*-value	OR	95%CI	*P*-value
Age > 50	1.913	0.79, 4.64	0.151	1.603 *1.930*	0.55, 4.67 *0.75, 4.92*	0.387 *0.169*
Male	1.774	0.61, 5.18	0.294	0.581 *0.848*	0.16, 2.16 *0.20, 2.97*	0.417 *0.796*
Alcohol intake > 40 g/d	1.081	0.28, 4.16	0.909	–	–	–
INH + RFP	0.469	0.16, 1.36	0.165	0.775 *0.538*	0.21, 2.86 *0.18, 1.64*	0.702 *0.275*
Latency < 1 month	0.667	0.21, 2.11	0.490	–	–	–
Hepatocellular DILI	0.689	0.27, 1.76	0.435	–	–	–
Cirrhosis	13.347	4.05, 43.98	0.000	11.484	3.16, 41.73	0.000
HBsAg-positive	3.284	1.26, 8.59	0.015	2.658 *3.232*	1.19, 5.58 *1.08, 9.64*	0.041 *0.035*

In addition to male gender, variables with *P* < 0.200 in the univariate analysis were included in the multivariate analysis. Adjusted statistical data were shown in italic font if the variable of cirrhosis was discarded due to its correlation with chronic HBV infection

Abbreviations: *INH* isoniazid, *RFP* rifampin, *DILI* drug-induced liver injury, *HBsAg* hepatitis B virus surface antigen

**Table 2:** Analyses of risk factors for the incidence of liver failure in the TB-HBV group (*n* = 58)

Factors	Univariate analysis			Multivariate analysis		
	OR	95%CI	*P*-value	OR	95%CI	*P*-value
Age > 50	1.306	0.43, 3.95	0.636	1.024	0.26, 3.99	0.973
Male	3.469	0.53, 22.80	0.195	1.199	0.14, 10.09	0.868
Alcohol intake > 40 g/d	0.429	0.06, 1.57	0.272	–	–	–
INH + RFP	0.533	0.15, 1.93	0.339	0.586	0.13, 2.61	0.483
Latency < 1 month	0.429	0.09, 1.94	0.272	–	–	–
Hepatocellular DILI	0.638	0.19, 2.15	0.467	–	–	–
Cirrhosis	9.545	7.7, 170.83	0.001	9.648	2.39, 38.87	0.001
HBV DNA > 20,000 IU/ml	1.045	0.34, 3.18	0.938	0.607	0.16, 2.33	0.466

Abbreviations: *TB* tuberculosis, *INH* isoniazid, *RFP* rifampin, *DILI* drug-induced liver injury, *HBV* hepatitis B virus

### Clinical outcomes related to liver injury

Of all patients, 24 (28.6%) had poor outcomes (Fig. 1), 22 of whom (91.7%) died during the 6-month follow-up or during hospitalization. Univariate analyses (Table 3) showed that age > 50 years old, cirrhosis and HBV co-infection were correlated to poor clinical outcomes. Male gender, alcohol intake > 40 g/d, anti-TB medications including both INH and RFP, latency of DILI < 1 month, and hepatocellular pattern of DILI had no correlations to poor clinical outcomes. Further multivariate analyses (Table 3) demonstrated that cirrhosis was an independent risk factor of poor outcomes (OR = 4.382, 95%CI: 1.35–14.21, *P =* 0.014), but HBV co-infection and age > 50 years old had borderline significance. If the variable of cirrhosis was discarded due to its correlation with chronic HBV infection, we found that both HBV co-infection (adjusted OR = 8.012, *P* = 0.010) and age > 50 years old (adjusted OR = 3.346, *P* = 0.027) were risk factors of poor clinical outcomes.

**Table 3:** Analyses of risk factors for poor clinical outcomes in all patients (*n* = 84)

Factors	Univariate analysis			Multivariate analysis		
	OR	95%CI	*P*-value	OR	95%CI	*P*-value
Age > 50	3.221	1.19, 8.70	0.021	2.859 *3.346*	0.93, 8.78 *1.15, 9.73*	0.067 *0.027*
Male	2.130	0.55, 8.21	0.272	–	–	–
Alcohol intake > 40 g/d	4.059	0.48, 33.94	0.196	4.199 *5.219*	0.44, 39.73 *0.58, 46.68*	0.211 *0.139*
INH + RFP	0.739	0.26, 2.14	0.740	–	–	–
Latency < 1 month	0.506	0.15, 1.70	0.270	–	–	–
Hepatocellular DILI	1.308	0.47, 3.66	0.609	–	–	–
Cirrhosis	7.588	2.57, 22.37	0.000	4.382	1.35, 14.21	0.014
HBV co-infection	7.333	1.58, 34.10	0.011	4.504 *8.012*	0.84, 24.05 *1.66, 38.66*	0.078 *0.010*

Only variables with P < 0.200 in the univariate analysis were included in the multivariate analysis. Adjusted statistical data were shown in italic font if the variable of cirrhosis was discarded due to its correlation with chronic HBV infection

Abbreviations: *INH* isoniazid, *RFP* rifampin, *DILI* drug-induced liver injury, *HBV* hepatitis B virus

In the TB-HBV group, 22 (37.9%) patients developed poor clinical outcomes (20 died, 1 deteriorated and 1 received an OLT), and the overall survival rate was 65.5%. Age > 50 years old, low PLT count, high TBIL level, HBV DNA > 20,000 IU/ml, severe coagulopathy (elevated PT level, PTA < 40% or INR > 1.5), cirrhosis and complications including HE, spontaneous bacterial peritonitis (SBP) and ascites were correlated with poor clinical outcomes (more details seen in Additional file 2). We also found that NAs as rescue therapy including LAM, ETV, LDT and ADV did not have a substantial influence on short-term clinical outcomes. Other therapies for liver failure including glucocorticoids, plasma exchange and allogeneic stem cell transfusion were administered in a few patients, and these treatments did not exert a significantly different impact on the prognosis. Univariate analyses (Table 4) indicated that age > 50 years old, cirrhosis, and HBV DNA > 20,000 IU/ml were correlated with poor clinical outcomes, while NAs as rescue therapy were not (OR = 1.071, 95%CI: 0.36–3.33, *P =* 0.905). Further multivariate analyses demonstrated that cirrhosis (OR = 6.320, 95%CI: 1.53–26.06, *P =* 0.011) and HBV DNA > 20,000 IU/ml (OR = 5.808, 95%CI: 1.37–24.64, *P =* 0.017) were risk factors for poor clinical outcomes.

**Table 4:** Analyses of risk factors for poor clinical outcomes in the TB-HBV group (*n* = 58)

Factors	Univariate analysis			Multivariate analysis		
	OR	95%CI	*P*-value	OR	95%CI	*P*-value
Age > 50	3.791	1.23, 11.69	0.020	3.317	0.91, 12.04	0.068
Male	2.625	0.27, 25.14	0.402	–	–	–
Alcohol intake > 40 g/d	5.069	0.58, 44.36	0.143	2.744	0.19, 40.15	0.461
INH + RFP	1.173	0.36, 3.80	0.790	–	–	–
Latency < 1 month	0.979	0.21, 4.57	0.978	–	–	–
Hepatocellular DILI	1.507	0.47, 4.80	0.488	–	–	–
Cirrhosis	4.76	1.44, 15.76	0.011	6.320	1.53, 26.06	0.011
HBV DNA > 20,000 IU/ml	3.800	1.16, 12.52	0.028	5.808	1.37, 24.64	0.017
NA treatment	1.071	0.36, 3.33	0.905	–	–	–

Only variables with P < 0.200 in the univariate analysis were included in the multivariate analysis

Abbreviations: *INH* isoniazid, *RFP* rifampin, *DILI* drug-induced liver injury, *HBV* hepatitis B virus, *NA* nucleos(t)ide analogue

**Fig. 2:**
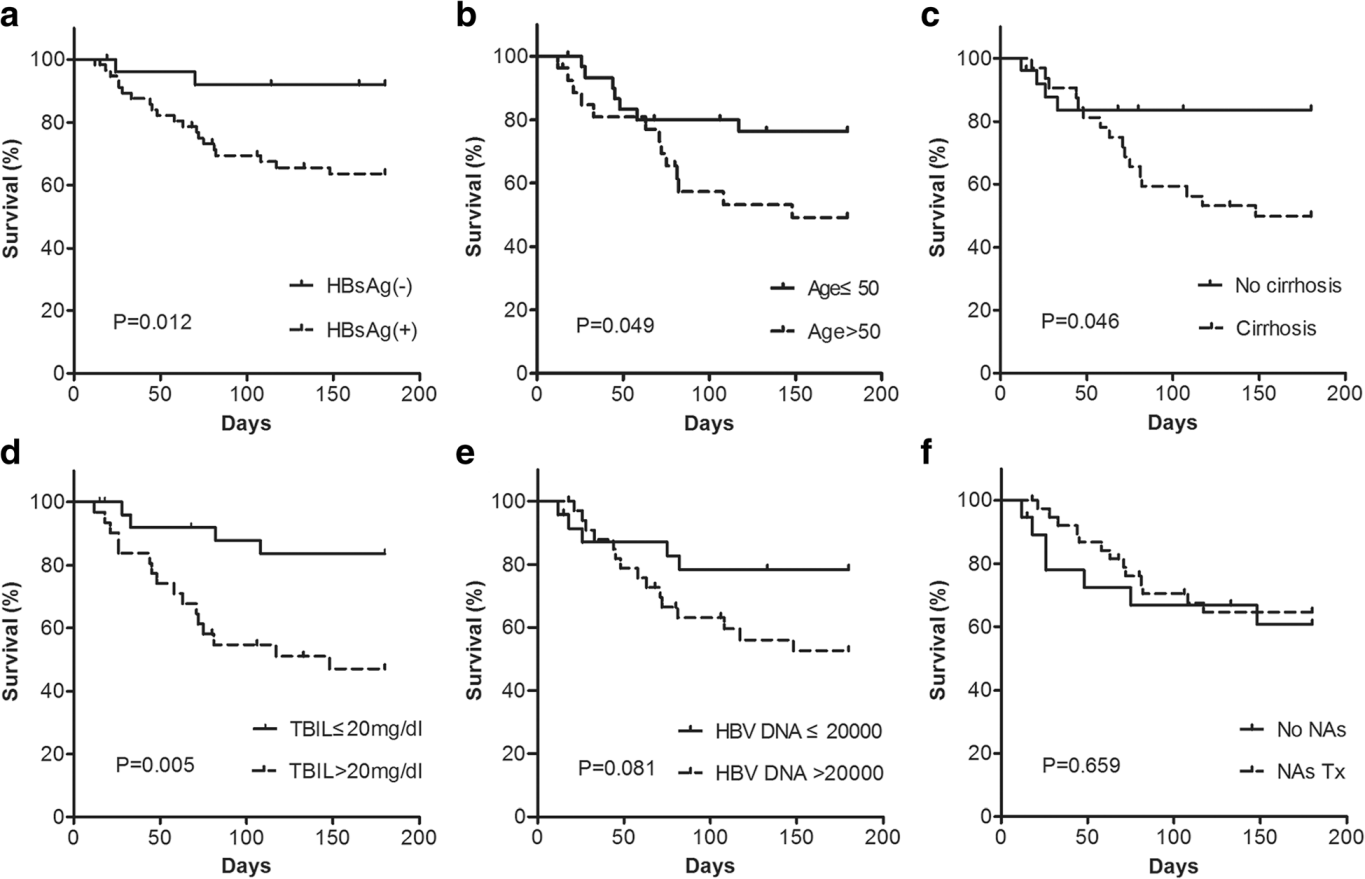
Kaplan-Meier curve for the survival analysis. Survival analysis in all patients showed that **a** HBV co-infection was an independent risk factor for death. Survival analyses in the TB-HBV group showed that **b** age > 50 years, **c** cirrhosis and **d** TBIL > 20 mg/dl were independent risk factors for death; **e** HBV DNA > 20,000 IU/ml had borderline statistical significance as risk factor for death. However, **f** NAs as rescue therapy were not able to reduce short-term death once liver failure had occurred

Kaplan-Meier survival analysis showed that patients in the TB-HBV group had a higher rate of death than those in the TB group (34.5% vs. 7.7%, *P =* 0.012, Fig. 2a). Further analyses of the TB-HBV group indicated that age > 50 years old (48.1% vs. 22.6%, *P =* 0.049, Fig. 2b), liver cirrhosis (50.0% vs. 15.4%, *P =* 0.046, Fig. 2c) and TBIL > 20 mg/dl (51.6% vs. 14.8%, *P =* 0.005, Fig. 2d) were significantly correlated with a higher rate of death due to anti-TB-related DILI. Moreover, there was a similar finding for high HBV DNA levels > 20,000 IU/ml (44.1% vs. 20.8%, *P =* 0.081, Fig. 2e) with borderline significance. The use of NAs as rescue therapy for anti-HBV treatment after the onset of DILI had no significant influences on the overall survival in this subgroup (33.3% vs. 36.8%, *P =* 0.659, Fig. 2f).

### HBV DNA at baseline and after anti-TB treatment

**Fig. 3:**
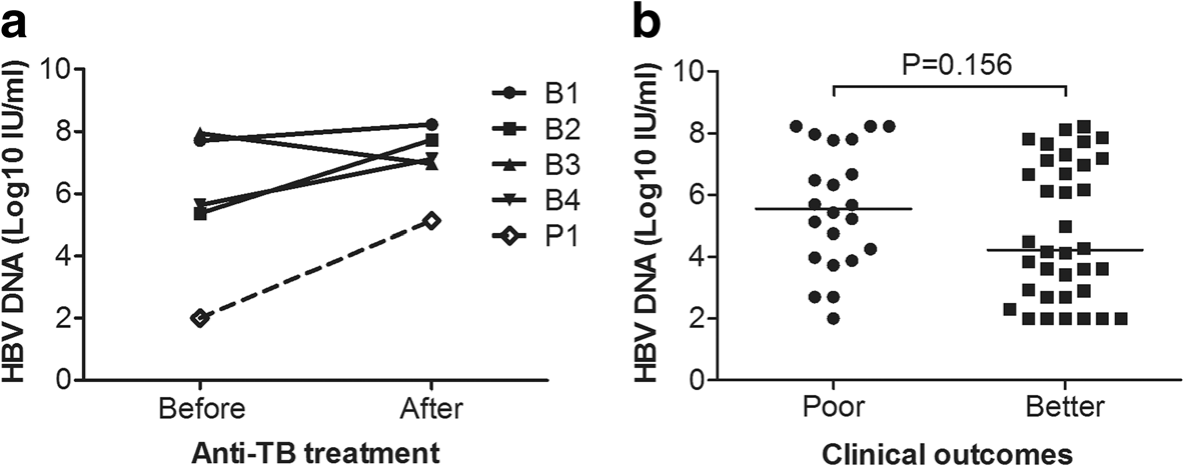
HBV DNA profiles in the TB-HBV group. **a** HBV DNA changes before and after anti-TB treatment. B1, B2, B3 and B4 (solid line) were patients with better clinical outcomes. P1 (dot line) indicated the patient who underwent a sharp increase in HBV DNA from 2.00 log to 5.13 log and subsequently died; **b** HBV DNA levels after the onset of liver injury and admission to our hospital in different outcome subgroups (Median [log_10_ IU/ml]: 5.55 vs. 4.24, *P =* 0.156)

All of the patients in the TB-HBV group received anti-TB treatment in other hospitals, and none of them received regular monitoring of liver function or HBV DNA. Only five patients had documented HBV DNA levels before anti-TB treatment had been initiated, and there was an increasing trend in HBV DNA levels after receiving anti-TB treatment. One patient exhibited a sharp increase in HBV DNA levels from 2.00 log to 5.13 log and consequently died, despite receiving anti-HBV treatment with ETV at 0.5 mg/d (Fig. 3a). Although all patients received HBV DNA quantification after being admitted to our hospital, most of their HBV DNA levels before anti-TB treatment were unknown, and overall dynamic changes in HBV DNA could not be available in this study. There were no significant differences in HBV DNA levels after admission to our hospital between the better and poor outcome subgroups (Median [log_10_ IU/ml]: 4.24 vs. 5.55, *P =* 0.156, Fig. 3b).

## Discussion

This study examined the rates of liver failure, poor clinical outcomes and survival in 84 TB patients with DILI. We found a higher incidence of liver failure and poor clinical outcomes in TB patients with HBV co-infection than in those with TB mono-infection. Age > 50 years, HBV co-infection and TBIL > 20 mg/dl were independently associated with poor clinical outcomes in patients who developed DILI due to anti-TB medications. The fact that NAs as rescue therapy after the initiation of DILI did not prevent further progression of liver failure suggests that early intervention with antiviral prophylaxis and regular monitoring of HBV replication may be crucial to the management of hepatotoxicity.

The risk of anti-TB drug-induced hepatotoxicity was higher in TB patients with chronic HBV co-infection compared to uninfected subjects in the current study as well as in others [17, 18]. Our data showed more severe DILI (Grade-4: 36.2% vs. 7.7%), more frequent liver failure (67.2% vs. 38.5%), more liver cirrhosis (55.2% vs. 11.5%) and poor clinical outcomes (37.9% vs. 7.7%) in the TB-HBV group, suggesting that HBV co-infection was a high risk factor for liver injury due to anti-TB medications. Published studies have indicated that most first-line anti-TB drugs induce hepatotoxicity via the formation of reactive metabolites, mediation of adaptive immune response, or bilirubin interference [19–21]. A pro-inflammatory environment induced by active HBV replication may accelerate the detoxification process and increase drug toxicities [22]. HBV DNA levels > 20,000 IU/ml seemed to be another independent risk factor of poor clinical outcomes. But we noticed that HBV DNA levels after the onset of anti-TB drug related DILI did not represent the real viral profiles prior to anti-TB treatment. Whether anti-TB medications reactivate or promote the replication of HBV remains unclear. Nevertheless, highly active HBV replication can lead to hepatitis flares, progressive liver fibrosis and cirrhosis even without a history of elevation or remarkable fluctuations in ALT [23]. Reactivation of HBV can also have significant clinical consequences including liver failure, which has a high mortality rate [24–26]. Unfortunately, most of these patients in this study did not have HBV DNA levels checked before their anti-TB treatment had been initiated. Only 5 patients had documented HBV DNA levels before anti-TB treatment, and 4 of them exhibited elevated HBV DNA to some extent.

According to the natural history of chronic HBV infection, more patients would be at risk of severe liver fibrosis and cirrhosis as their ages advanced [23]. In the TB-HBV group, patients had an average 10-year history of chronic HBV infection (perhaps even longer) and had a mean age of approximately 49 years. We believe that this was the main reason accounting for the more frequent cirrhosis in co-infected TB/HBV patients. Two TB mono-infected patients were considered to have liver cirrhosis, which might be attributed to excessive alcohol intake (> 40 g/d).

In addition to HBV DNA level, age was also associated with an increased risk of poor outcomes. In a cohort of over 3000 TB patients receiving INH monotherapy, the incidence of DILI was higher in those aged 50 years or older [27]. The severity of hepatotoxicity and consequent mortality has also been reported to be higher after the age of 50 years old [21]. Consistent with these reports, our data from multivariate analyses indicated that advanced age (> 50 years) was an independent risk factor of poor outcomes and overall survival in patients with DILI due to first-line anti-TB medications regardless of HBV co-infection. However, most cases of chronic HBV infection in highly endemic areas are due to HBV infection at birth or during the first year of life, and cirrhosis may occur at early ages. Therefore, it is not surprising that poor outcomes would be much more likely in TB/HBV co-infected patients in an environment of chronic inflammation or hepatocyte necrosis. Moreover, individuals with chronic hepatitis B who are aged 40 years or older should be considered frequently for HBV DNA testing or liver biopsy or for the initiation of anti-viral therapy in some cases, according to guidelines from international associations [28].

In our investigation, anti-TB regimens including the first-line medications of INH, RFP plus PZA and/or EMB accounted for a major component of liver injury in both TB and TB-HBV groups. The patterns of DILI were similarly distributed according to hepatocellular, cholestatic and mixed subtypes. Anti-TB drug related DILI often developed 1 to 3 months after anti-TB treatment, as reported elsewhere [29]. Our data were in accordance with this duration, but the difference between TB mono-infected patients and TB/HBV co-infected patients was not statistically significant. Nevertheless, it seemed that chronic HBV co-infection did not exert an impact on the latency of DILI onset. In a study by WS Kim et al. [29], they found that the mean onset time of DILI in patients with and without chronic HBV and/or HCV co-infection differed somewhat but not significantly (Mean [days]: 83.3 vs. 43.9, *P* = 0.056). We noticed that almost all patients had not received regular liver function monitoring after they had initiated anti-TB treatment and were transported to our hospital when developing severe liver injury and even after liver failure had developed. The actual onsets of DILI were not well known or established.

Prompt withdrawal of the offending medications and administration of NAs including LAM, ADV, LDT and ETV for 39 TB patients HBV DNA levels > 2000 IU/ml could not slow the progression of liver damage. Similar rates of poor outcomes were observed between patients with and without NAs rescue therapy in the current study; 20 TB patients with HBV co-infection still died. This implied that once liver injury had developed, the use of NAs as rescue therapy for anti-HBV treatment seemed unable to affect short-term survival in this setting. Another retrospective investigation of a small sample found that baseline liver function test abnormality and HBV DNA levels > 2000 IU/ml were independent risk factors for the development of DILI in TB patients with HBV co-infection [10]. Our study was in accordance with these findings. Poor clinical outcomes might due to poor hepatocellular regeneration and pre-existing liver fibrosis or cirrhosis to some extent [30, 31].

We recommend that NAs should be administered for patients with active HBV DNA replication before anti-TB treatment to prevent the development of liver failure. Regarding the current clinical practice guidelines for chronic hepatitis B management [3, 23], patients with malignant tumors who receive chemotherapy should be regularly monitored for ALT and HBV DNA levels and treated with NA therapy upon confirmation of HBV reactivation prior to ALT elevation. Huang et al. [32] compared the use of prophylactic ETV before and after rituximab-based chemotherapy and found that early intervention with anti-HBV therapy can effectively prevent HBV reactivation. A recent randomized open-label, phase 3 study from China compared ETV with LAM for the treatment of HBsAg-positive patients with low viral loads receiving R-CHOP chemotherapy. The results revealed significantly lower rates of HBV-related hepatitis, HBV reactivation and chemotherapy disruption in the ETV group [33].

It is important to perform HBV screening before starting anti-TB therapy, ideally at the time of diagnosis of the condition requiring anti-TB treatment. In some cases, in which an HBV core-positive profile is only reflected after HBV infection (anti-HBcAb-positive and HBsAg-negative), so-called occult HBV infection [34], the positive results could also reflect passively acquired antibodies from recent blood products or may rarely be due to nonspecific reactivity or HBsAg mutant infection. Additional testing is critical to clarify patients’ true HBV status. This means that there needs to be close collaboration between virology and clinical teams to ensure that all test results are interpreted accurately.

We acknowledge that this study is limited by its small number of patients, its retrospective nature, and the lack of overall information on HBV DNA levels before and at the onset of DILI. However, the results of this study highlight the importance of HBV DNA and liver function monitoring in TB patients with chronic HBV co-infection who plan to receive anti-TB treatment, as well as the fine balance between prompt initiation of anti-HBV intervention versus avoidance of unnecessary antiviral prophylaxis.

## Conclusions

In this retrospective investigation, we found that patients in the TB-HBV group were more susceptible to developing liver failure and having poor outcomes during anti-TB treatment compared to those in the TB group. Advanced age, cirrhosis and severe hyperbilirubinemia were independent risk factors for the incidence of death in the TB-HBV group, and HBV DNA > 20,000 IU/ml had borderline significance. NAs as rescue therapy were not able to reduce short-term death once liver failure had occurred. These findings suggest that regular monitoring of liver function and HBV DNA level during anti-TB treatment is indispensable for TB patients with chronic HBV co-infection, and anti-HBV treatment should be considered in those with high HBV DNA levels before anti-TB treatment to prevent them from developing liver failure.

## Additional files

Additional file 1: **Table S1.** Demographics and clinical characteristics between TB group and TB-HBVgroup. It showed that more patients in the TB-HBV group experienced severe hyperbilirubinemia (Median of TBIL [μmol/L]: 391.4 vs. 145.8, *P =* 0.007), cirrhosis (55.2% vs. 7.7%, *P =* 0.000), Grade-4 DILI (36.2% vs. 7.7%, *P =* 0.015), liver failure (67.2% vs. 38.5%, *P =* 0.013) and had poor clinical outcomes (37.9% vs. 7.7%, *P =* 0.005), compared with those in the TB group. (DOCX 23 kb)

Additional file 2: **Table S2.** Demographics and characteristics of patients in TB-HBV group with different clinical outcomes. Compared with those with better clinical outcomes, the proportions of patients with advanced age (Mean [years]: 53.9 vs. 45.5, *P =* 0.017; age > 50 years old: 63.6% vs. 36.1%, *P =* 0.041), severe hyperbilirubinemia (Median of TBIL [μmol/L]: 478.8 vs. 251.0, *P =* 0.000), cirrhosis (77.3% vs. 41.7%, *P =* 0.008) and HBV DNA > 20,000 IU/L (77.3% vs. 47.2%, *P =* 0.024) in the TB-HBV group were significantly higher. (DOCX 22 kb)

## Abbreviations

ADV: Adefovir
ALP: Alkaline phosphatase
ALT: Alanine aminotransferase
AST: Aspartate aminotransferase
CI: Confidence interval
DILI: Drug-induced liver injury
EMB: Ethambutol
ETV: Entecavir
HBsAg: Hepatitis B virus surface antigen
HBV: Hepatitis B virus
HE: Hepatic encephalopathy
INH: Isoniazid
INR: International normalized ratio
LAM: Lamivudine
LDT: Telbivudine
NA: Nucleos(t)ide analogue
OLT: Orthotopic liver transplantation
OR: Odds ratio
PLT: Platelet
PT: Prothrombin time
PTA: Prothrombin time activity
PZA: Pyrazinamide
RFP: Rifampin
SBP: Spontaneous bacterial peritonitis
TB: Tuberculosis
TBIL: Total bilirubin
ULN: Upper limit of normal
