# Supplementary material for: Co-infection with hepatitis B virus among tuberculosis patients is associated with poor outcomes during anti-tuberculosis treatment
Source: BMC Infect Dis. 2018 Jul 3;18:295. doi: 10.1186/s12879-018-3192-8 (PMC6029116; doi:10.1186/s12879-018-3192-8)
Supplement: Supplementary file 1 — Table S1. Demographics and clinical characteristics between TB group and TB-HBVgroup. It showed that more patients in the TB-HBV group experienced severe hyperbilirubinemia (Median of TBIL [μmol/L]: 391.4 vs. 145.8, P = 0.007), cirrhosis (55.2% vs. 7.7%, P = 0.000), Grade-4 DILI (36.2% vs. 7.7%, P = 0.015), liver failure (67.2% vs. 38.5%, P = 0.013) and had poor clinical outcomes (37.9% vs. 7.7%, P = 0.005), compared with those in the TB group. (DOCX 23 kb) [file 12879_2018_3192_MOESM1_ESM.docx]

**Table S1** Demographics and clinical characteristics between TB group and TB-HBV group

| Factors | TB group  (n=26) | TB-HBV group  (n=58) | *P*-value |
| --- | --- | --- | --- |
| Age (years old) | 48.6 ± 14.8 | 49.2 ± 12.8 | 0.850 |
| Male | 14(53.9) | 53(91.4) | 0.000 |
| Alcohol intake >40g/d | 2(7.7) | 7(12.1) | 0.827 |
| Sites of TB  Pulmonary  Extrapulmonary | 17(65.4)  9(34.6) | 48(82.8)  10(27.2) | 0.078 |
| Anti-TB regimens  INH + RFP + EMB/PZA/both  INH + Ofloxacin/Levofloxacin | 22(84.6)  4(15.4) | 41(70.7)  17(29.3) | 0.173 |
| Latency of DILI (days) | 79.5(45.0-112.0) | 84.0(49.5-126.5) | 0.462 |
| Hospital stays (days) | 23.0(8.5-33.5) | 13.5(7.5-33.5) | 0.447 |
| Patterns of DILI  Hepatocellular  Cholestatic  Mixed | 17(65.4)  6(23.1)  3(11.5) | 39(67.2)  8(13.8)  11(19) | 0.465 |
| Creatinine_max_(μmol/L) | 82.6 ± 52.0 | 90.3 ± 53.6 | 0.540 |
| WBC_max_(×10^9^/L) | 8.4 ± 4.3 | 6.8 ± 3.4 | 0.090 |
| PLT_min_(×10^9^/L) | 188.5 ± 87.6 | 133.8 ± 53.6 | 0.001 |
| Liver function tests |  |  |  |
| ALT_max_(U/L) | 548.0(104.3-854.8) | 285.0(152.3-674.0) | 0.416 |
| AST_max_ (U/L) | 300.0(131.3-536.0) | 195.0(114.0-594.5) | 0.731 |
| TBIL_max_(μmol/L) | 145.8(37.7-387.6) | 391.4(165.3-557.4) | 0.007 |
| ALP_max_ (U/L) | 123.5(91.0-169.3) | 116.0(94.3-153.0) | 0.552 |
| Coagulopathy |  |  |  |
| PT_max_ (sec) | 25.2 ± 14.3 | 27.5 ± 13.1 | 0.474 |
| PTA <40% | 10(38.5) | 30(51.7) | 0.261 |
| INR_max_ | 2.4 ± 1.80 | 2.7 ± 1.7 | 0.540 |
| INR >1.5 | 12(46.2) | 45(77.6) | 0.004 |
| Complications |  |  |  |
| HE | 4(15.4) | 20(34.5) | 0.073 |
| HRS | 0(0) | 7(8.3) | 0.155 |
| SBP | 4(15.4) | 19(32.8) | 0.099 |
| Hemorrhage | 0(0) | 2(3.4) | 1.000 |
| Lung infection | 1(3.8) | 11(19.0) | 0.135 |
| Ascites | 4(15.4) | 28(48.3) | 0.004 |
| Cirrhosis | 2(7.7) | 32(55.2) | 0.000 |
| Severity of DILI  Grade 1  Grade 2  Grade 3  Grade 4 | 5(19.2)  10(38.5)  9(34.6)  2(7.7) | 5(8.6)  10(17.2)  22(37.9)  21^†^(36.2) | 0.015 |
| Liver failure | 10(38.5) | 39(67.2) | 0.013 |
| Clinical outcomes  Better  Recovered/Improved  Poor  Exacerbated/Died | 24(92.3)  15/9  2(7.7)  0/2 | 36(62.1)  16/20  22(37.9)  2/20 | 0.005 |

^†^including 20 patients died and 1 patient received orthotopic liver transplantation.

Continuous variables were expressed as mean ± standard deviation or median (interquartile range), and categorical variables were expressed as frequency (%).

Abbreviations: TB, tuberculosis; HBV, hepatitis B virus; INH, isoniazid; RFP, rifampin; PZA, pyrazinamide; EMB, ethambutol; DILI, drug-induced liver injury; WBC, white blood cell; PLT, platelet; ALT, alanine aminotransferase; AST, aspartate aminotransferase; TBIL, total bilirubin; GGT, gama-glutamyl transferase; ALP, alkaline phosphatase; PT, prothrombin time; PTA, prothrombin time activity; INR, international normalized ratio; HE, hepatic encephalopathy; HRS, hepatic renal syndrome; SBP, spontaneous bacterial peritonitis.
