# Supplementary material for: Co-infection with hepatitis B virus among tuberculosis patients is associated with poor outcomes during anti-tuberculosis treatment
Source: BMC Infect Dis. 2018 Jul 3;18:295. doi: 10.1186/s12879-018-3192-8 (PMC6029116; doi:10.1186/s12879-018-3192-8)
Supplement: Supplementary file 2 — Table S2. Demographics and characteristics of patients in TB-HBV group with different clinical outcomes. Compared with those with better clinical outcomes, the proportions of patients with advanced age (Mean [years]: 53.9 vs. 45.5, P = 0.017; age > 50 years old: 63.6% vs. 36.1%, P = 0.041), severe hyperbilirubinemia (Median of TBIL [μmol/L]: 478.8 vs. 251.0, P = 0.000), cirrhosis (77.3% vs. 41.7%, P = 0.008) and HBV DNA > 20,000 IU/L (77.3% vs. 47.2%, P = 0.024) in the TB-HBV group were significantly higher. (DOCX 22 kb) [file 12879_2018_3192_MOESM2_ESM.docx]

**Table S2** Demographics and characteristics of patients in TB-HBV group with different clinical outcomes

| Factors | Poor outcome  (n=22) | Better outcome  (n=36) | *P*-value |
| --- | --- | --- | --- |
| Age (years)  ≤50  >50 | 53.9 ± 12.9  8(36.4)  14(63.6) | 45.5 ± 12.4  23(63.9)  13(36.1) | 0.017  0.041 |
| Male | 21(95.5) | 32(88.9) | 0.702 |
| Alcohol intake>40g/d | 0(0) | 7(19.4) | 0.073 |
| Latency of DILI (days) | 82.5(60.0-123.0) | 84.5(38.8-138.5) | 0.866 |
| Hospital stays (days) | 8.5(3.0-22.3) | 21.0(11.0-54.0) | 0.003 |
| HBV infection (years) | 7.0(0-20.0) | 10.0(0-20.0) | 0.851 |
| Types of DILI  Hepatocellular  Cholestatic  Mixed | 16(72.7)  4(18.2)  2(9.1) | 23(63.9)  4(11.1)  9(2.5) | 0.263 |
| WBC_max_ (×10^9^/L) | 7.8 ± 4.5 | 6.3 ± 2.4 | 0.154 |
| PLT_min_ (×10^9^/L) | 115.0 ± 55.1 | 144.8 ± 50.3 | 0.044 |
| Creatinine_max_ (μmol/L) | 104.3 ± 67.5 | 81.8 ± 41.9 | 0.169 |
| HBeAg-positive | 4(18.2) | 8(22.2) | 0.972 |
| HBV DNA (log_10_ IU/ml)  ≤20000 IU/ml  >20000 IU/ml | 5.55(2.0-8.23)  5(22.7)  17(77.3) | 4.24(2.0-8.23)  19(52.8)  17(47.2) | 0.156  0.024 |
| Liver function tests |  |  |  |
| ALT_max_ (U/L) | 362.5(109.5-778.3) | 207.5(155.3-650.0) | 0.785 |
| AST_max_ (U/L) | 320.5(86.0-775.3) | 181.0(124.0-503.0) | 0.481 |
| TBIL_max_ (μmol/L) | 478.8(364.3-653.9) | 251.0(84.4-463.3) | 0.000 |
| GGT_max_ (U/L) | 108.0(45.3-137,8) | 123.5(88.0-174.5) | 0.082 |
| ALP_max_ (U/L) | 111.5(97.8-137.0) | 117.5(92.8-160.5) | 0.671 |
| Coagulopathy |  |  |  |
| PT_max_ (sec) | 39.8 ± 12.2 | 20.0 ± 6.2 | 0.000 |
| PTA_min_ <40% | 22(100) | 8(22.2) | 0.000 |
| INR_max_ >1.5 | 22(100) | 23(63.9) | 0.004 |
| Complications |  |  |  |
| HE | 17(77.3) | 3(8.3) | 0.000 |
| Hemorrhage | 1(4.5) | 1(2.8) | 1.000 |
| HRS | 4(18.2) | 3(8.3) | 0.483 |
| SBP | 12(54.5) | 7(19.4) | 0.006 |
| Ascites | 16(72.7) | 12(33.3) | 0.004 |
| Lung infection | 5(22.7) | 6(16.7) | 0.821 |
| Cirrhosis | 17(77.3) | 15(41.7) | 0.008 |
| Rescuing anti-HBV  NAs^*^  None | 15(68.2)  7(31.8) | 24(66.7)  12(33.3) | 0.905 |
| Glucocorticoids | 1(2.8) | 0(0) | 1.000 |
| Plasma exchange | 2(5.6) | 3(13.6) | 0.357 |
| Stem cell transfusion | 2(5.6) | 4(18.2) | 0.187 |

NAs^*^: Including lamivudine (4 *vs.* 2), adefovir (2 *vs.* 0), telbivudine (1 *vs*. 1), and entecavir (17 *vs.* 12) in better *vs.* poor outcome groups, respectively.

Continuous variables were expressed as mean ± standard deviation or median (interquartile range), and categorical variables were expressed as frequency (%).

Abbreviations: TB, tuberculosis; HBV, hepatitis B virus; INH, isoniazid; RFP, rifampin; PZA, pyrazinamide; EMB, ethambutol; DILI, drug-induced liver injury; WBC, white blood cell; PLT, platelet; ALT, alanine aminotransferase; AST, aspartate aminotransferase;TBIL, total bilirubin; GGT, gama-glutamyl transferase; ALP, alkaline phosphatase; PT, prothrombin time; PTA, prothrombin time activity; INR, international normalized ratio; HE, hepatic encephalopathy; HRS, hepatic renal syndrome; SBP, spontaneous bacterial peritonitis; HBeAg, hepatitis B virus e antigen; NAs, nucleos(t)ide analogues.
